# Elevated Liver Enzymes and Genotype I of T. *gondii* Among Liver Cancer Patients: Evidence of a Potential Link

**DOI:** 10.31557/APJCP.2026.27.1.361

**Published:** 2026-01-22

**Authors:** Nawadir H. Jasim, Ali B. M. Al-Waaly

**Affiliations:** *Department of Biology, College of Sciences, University of Al-Qadisiyah, Al-Qadisiyah 58001, Iraq. *

**Keywords:** Toxoplasma gondii, PCR-RFLP, GRA6 gene, Nested-PCR and Genotyping

## Abstract

**Objective:**

*Toxoplasma gondii* is an intracellular protozoan that may disrupt the traditional cell barriers against cancer, allowing the accumulation of oncogenic mutations over time. Our research aimed to explore the relationship between *T. gondii* infection and liver cancer development.

**Methods:**

The present study, conducted in the city of Nasiriya, Iraq, involved 80 blood samples collected from individuals aged 18 to 70 years, of both sexes. The samples were divided into two groups: 40 from patients diagnosed with liver cancer and 40 from healthy individuals. All samples were tested using ELISA to detect anti-*Toxoplasma gondii *antibodies (IgG and IgM).

**Results:**

The results showed that 21 liver cancer patients and 14 healthy individuals tested positive for *T. gondii*. Furthermore, liver enzyme levels (ALT, AST, and ALP) were assessed in all participants. The findings revealed a notable elevation in enzyme levels among liver cancer patients co-infected with *T. gondii*, compared to both non-infected liver cancer patients and the healthy control group. Using PCR, the *B1* gene was amplified to confirm infection in selected samples. Ten B1-positive samples (5 from liver cancer and 5 from control) were tested for the *GRA6* gene using nested PCR. DNA was extracted with a commercial kit, and amplification was performed using specific primers. Genotyping was conducted via PCR-RFLP targeting the *GRA6* gene, using the MseI enzyme to distinguish *T. gondii* strains based on fragment sizes. RFLP analysis using MseI differentiated genotypes I, II, and III.

**Conclusion:**

This study genotyped *Toxoplasma gondii *in liver cancer patients using nested-PCR and RFLP targeting the *GRA6* gene. Genotype I predominated among liver cancer patients, whereas control Genotypes II was predominance in the control group.

## Introduction

*Toxoplasma gondii *is a parasite with a distinct life cycle that involves both asexual and sexual reproduction. The sexual cycle of the only species in the genus Toxoplasma, *T. gondii*, is exclusive to felines [1]. Human infection mainly occurs via ingesting food or water contaminated with the parasite cyst or oocyst stage. Vertical transmission of *T. gondii* infection can also occur in several host species [2–4]. Also, infection via blood transfusion and organ transplantation are other possible routes of transmission [4, 5]. Healthy people usually exhibit no clinical symptoms in response to *T. gondii* infection. However, individuals with immune deficiency, such as those with AIDS, can exhibit serious symptoms, such as headache and seizures [6, 7].

In immunocompetent individuals, Toxoplasma infection often passes unnoticed leaving the person immune against reinfection. Conversely, it causes fulminant life threatening disseminated disease in the immunosuppressed individuals including cancer patients [8]. Liver cancer (LC) is a prevalent malignant tumor and a major contributor to cancer-related mortality worldwide [9]. The primary types of liver cancer include hepatocellular carcinoma (HCC), cholangiocarcinoma, mixed-type cancer, and hemangioma. Among these, hepatocellular carcinoma accounts for more than 80% of cases. HCC originates from hepatocytes, which are the primary functional cells of the liver. It is often associated with chronic liver diseases such as viral hepatitis, alcoholic liver disease, and non-alcoholic fatty liver disease. The incidence of LC has been steadily increasing in many regions, presenting a significant public health challenge [10, 11].

The most common biomarkers used to evaluate liver injury are alanine aminotransferase (ALT), aspartate aminotransferase (AST), alkaline phosphatase (ALP), glutamyl transpeptidase (GGT), total bilirubin (TBIL), and lactate dehydrogenase (LDH). The levels of TBIL may correlate with the overall liver dysfunction, while ALP level is an indication of biliary damage, and high AST/ALT concentrations may indicate hepatocyte necrosis [12].

Worldwide studies have shown that *T. gondii* possesses significant genetic and phenotypic diversity; at present, three main lineages (Types I-III) are described, which vary in virulence and mortality for laboratory mice [13]. To determine *T. gondii* infection using sero-diagnosis, which could highly be useful to differentiate chronic and acute disease, are available [14]. The *T. gondii* type I strain is classified as highly virulent, leading to widespread parasite dissemination and lethal infection in mice (100% cumulative mortality). In contrast, mouse mortality and tachyzoite dissemination induced by type II or III strains are considerably lower (30%), with type III strains generally being considered a virulent for mice [15].

Using the PCR-RFLP assay with the Tru1I (MseI) enzyme, the dense granule protein gene (*GRA6* gene) can differentiate between three distinct *T. gondii* genotypes and functions as a single copy gene with a greater polymorphism rate than other markers [16]. With a high rate of DNA polymorphism, the *GRA6* gene is frequently employed as one of the best markers for assessing *T. gondii*’s genetic diversity since it can distinguish between the three distinct genetic types and some of the parasite’s atypical genotypes. Additionally, the isolates with the high genetic diversity *GRA6* gene have more distinct characteristics than those with the *B1* gene [17, 18]. The present study aims to identify the genotypes of *Toxoplasma gondii *in patients with liver cancer, as well as to investigate the potential association between *T. gondii* infection and the development of hepatocellular carcinoma.

## Materials and Methods

### Sample collection

A total of 40 samples were collected from patients suffering from liver cancer in addition to 40 samples from healthy persons as a control, during the period from January 2024 to December 2024. Initially, these samples were screened for *Toxoplasma gondii *infection using the ELISA technique. 35 of 80 were sero-positive for ELISA test. Subsequently, ELISA-positive samples were all subjected to confirmatory testing by detecting the *B1* gene using the PCR technique (Data not shown). Afterward, 10 samples (5 samples from liver cancer and 5 samples for control) of *B1* gene-positive samples were subjected to confirmatory testing by detecting the *GRA6* gene using the nested PCR technique. Informed consent was obtained from all individual participants included in the study. The study was approved by research committee of the medical ethics unit, University of Al-Qadisiyah.

### Liver Function Tests

Liver enzymes (AST, ALT and ALP) were measured using an automated chemistry analyzer according to the manufacturer’s instructions (Mindray, China).

### DNA extraction

Genomic DNA was extracted from the positive samples using a commercial kit (gSYNC™ DNA Extraction Kit) provided by (Geneaid Biotech LTD, Korea) and following the manufacturer’s instructions. The purity of the extracted DNA was then assessed using a NanoDrop spectrophotometer (Thermo Scientific, USA). Extracted DNA was kept at -20 C for further genetic analysis.

*Nested-PCR amplification targeted GRA6 gene*

A 10 positive samples of *B1* gene were randomly chosen and then subjected to further molecular analysis through nested polymerase chain reaction (nPCR) targeting the *GRA6* gene. Two pairs of primers were employed in this procedure: GRA6-FO1 (5′-GGCAAACAAAACGAAGTG-3′) and GRA6-RE1 (5′-CGACTACAAGACATAGAGTG-3′) for the first round of amplification, followed by GRA6-F1x (5′-GTAGCGTGCTTGTTGGCGAC-3′) and GRA6-R1x (5′-TACAAGACATAGAGTGCCCC-3′) for the second round [19–22].

The initial PCR reaction was carried out in a total volume of 25 µL, consisting of 10 µL of 2X PCR master mix (Promega Corporation, USA), 1 µL of each primer (forward and reverse), 5 µL of DNA template, and 8 µL of nuclease-free water. The second round of amplification was performed using the same total volume and reagent concentrations; however, the template used was 5 µL of the first-round PCR product. The thermal cycling conditions for both rounds included an initial denaturation at 95°C for 5 minutes, followed by 40 cycles of denaturation at 95°C for 30 seconds, annealing 55 °C for 30 seconds and extension at 72°C for 1 minute, and a final extension at 72°C for 6 minutes. The annealing temperature was 55°C for the first round and 58°C for the second round. Amplified products were then analyzed using 2% agarose gel electrophoresis to visualize the nPCR results.

### PCR-RFLP for strain detection

To genetically characterize *Toxoplasma gondii *strains, PCR-restriction fragment length polymorphism (PCR-RFLP) analysis targeting the *GRA6* gene was performed using the MseI restriction enzyme (New England Biolabs, UK). This method enables the differentiation among *T. gondii* genotypes based on specific digestion patterns of nested PCR (nPCR) amplicons. The MseI enzyme cleaves the amplified GRA6 fragments into distinctive sizes: 168 and 544 bp for type I, 75 and 623 bp for type II, and 97 and 544 bp for type III strains. The enzymatic digestion was conducted according to the manufacturer’s protocol by incubating the enzyme with the nPCR products at 37 °C for 4 hours. Post-incubation, the digested fragments were resolved on a 2% agarose gel via electrophoresis to verify enzymatic activity and to determine the corresponding allelic profiles of the *T. gondii* strains.

### Statistical Analysis

Statistical analysis was performed with SPSS version 21 (IBM Corporation, USA). Descriptive statistics for categorical data were expressed as frequency and percentage. ANOVA test is used to compare between results. p-value of ≤ 0.05 was considered as the level of significant.

## Results

### Prevalence Toxoplasma gondii infection in patients and control

The current study included 40 samples from liver cancer patients, in addition to 40 samples from healthy individuals as a control group. The samples were examined for the presence of *T. gondii* parasite using the ELISA test. The results revealed 21 positive infections (53%) in the liver cancer group, while the control group had 14 positive samples (35%). Statistical analysis indicated a significantly higher prevalence of *T. gondii* in liver cancer patients than in the control group (p<0.05) (Table 1).

### Effect of T. gondii Co-infection on liver function markers

Table (2) illustrates the mean concentrations of liver enzymes in samples from liver cancer patients co-infected and not co-infected with Toxoplasma gondii, as well as in control groups (both infected and uninfected with *T. gondii*). The results showed that the mean levels of (ALT, AST and ALP) were (76.370, 82.710 and 183.756), respectively, in liver cancer patients positive for *T. gondii*, while the mean levels were (67.010, 70,410 and 167.139) respectively, in patients negative for *T. gondii* infection. A statistically significant difference was observed (p<0.05). Additionally, when comparing healthy individuals infected with *T. gondii* to uninfected healthy individuals, a statistically significant elevation (p<0.05) was observed in the levels of (ALT, AST and ALP).

### Nested-PCR for the detection of GRA6 gene

For the GRA6 marker on chromosome X, we employed RFLP-nPCR to give a highly sensitive genotyping of *T. gondii*. The variation in this gene was identified by the GRA6 nPCR marker. Using MseI to digest the amplified GRA6 product allowed for the differentiation of genotypes I, II, and III. As a result, using different RFLP patterns, the GRA6 marker can clearly identify all three genotypes.

To reinforce the reliability of detecting *T. gondii* and to confirm the positive findings based on *B1* gene amplification (data not shown), an additional diagnostic assay was performed using a Nested-PCR approach targeting the *GRA6* gene. This gene encodes one of the dense granule proteins expressed during the intracellular phase of the parasite’s life cycle and is often utilized as a marker for active infection or genotyping purposes.

A total of five randomly selected B1-positive samples were obtained from each of the following patient groups: those with liver cancer and healthy individuals. These samples were subjected to Nested-PCR using specific primers for the *GRA6* gene.

**Figure 1:**
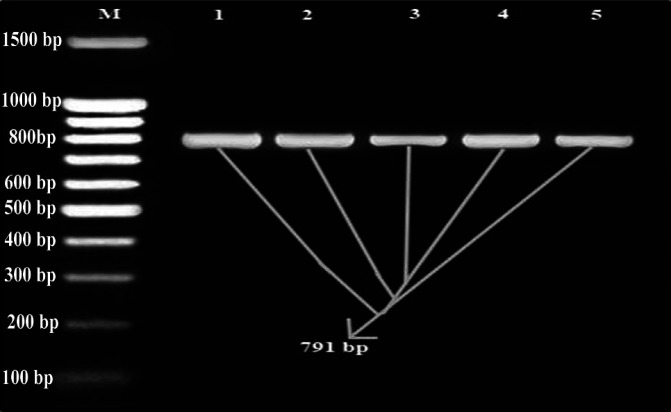
Molecular Confirmation of *T. gondii* *GRA6* Gene in Patients with Liver Cancer. Electrophoretic profile of nested-PCR products for the *GRA6* gene in blood samples from liver cancer patients. All samples (lanes 1-5) show distinct bands at 791 bp, reinforcing evidence of T. gondii presence in individuals with liver cancer. M: 100 bp ladder.

**Figure 2:**
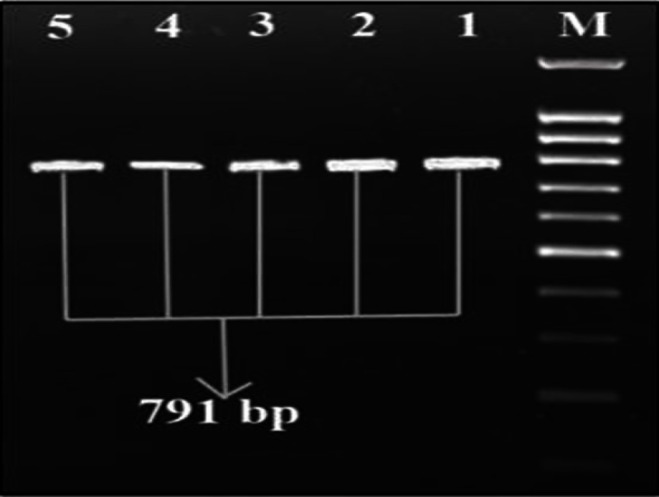
Detection of *GRA6* Gene in Control Group via Nested-PCR. Nested-PCR gel image showing *GRA6 *gene amplification in asymptomatic control subjects. All tested samples (lanes 1-5) revealed positive bands at 791 bp, aligning with previous B1 gene results. M: 100 bp ladder.

In the Figure 1, which represents the results from liver cancer patients, all five lanes (1-5) exhibited a distinct amplification band at the expected molecular size of 791 base pairs (bp). This confirms the presence of the *GRA6* gene sequence in each of the analyzed samples. Also, five samples from individuals in the control group were examined using Nested-PCR to detect the *GRA6* gene of Toxoplasma gondii. As shown in the Figure 2, all wells (1-5) revealed a distinct amplification band at the expected molecular size of 791 base pairs (bp), indicating positive results in all tested samples.

### Genotyping of T. gondii

The genotype of Toxoplasma samples was determined by utilizing RFLP-PCR to assess *Toxoplasma gondii *genotypes after gene-specific amplification of the GRA6 product was digested by restriction enzymes. Five DNA samples from patients diagnosed with liver cancer, previously confirmed to be positive for *Toxoplasma gondii *via GRA6 nested-PCR, were subjected to enzymatic digestion using the MseI restriction enzyme for genotyping analysis.

**Figure 3:**
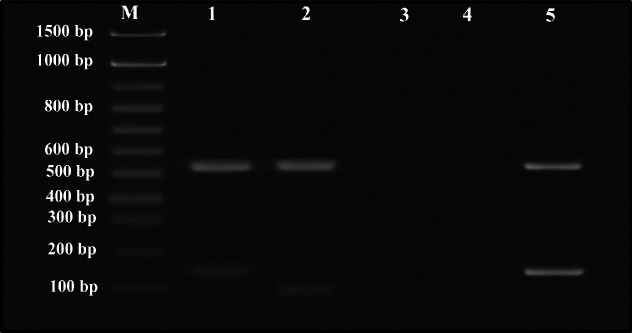
Representative Gel Image of* GRA6* Gene Digestion by MseI in Liver Cancer Patient Samples. The figure shows MseI digestion patterns of *GRA6* nested-PCR products from five liver cancer patient samples. Type I (lanes 1 and 5 with 168 and 544 bp) and Type III (lane 2 with 97 and 544 bp) genotypes were detected; Type II was absent. M; molecular marker (100 bp).

Gel electrophoresis of the digested products revealed genotypic variation in three of the five samples. Type I genotype was detected in two isolates, characterized by digestion bands of approximately 544 bp and 168 bp, which matches known banding patterns for this genotype. Type III was identified in one isolate, producing fragments of 544 bp and 97 bp. Notably, Type II was not detected in any of the samples from this group. These findings suggest a possible predominance of more virulent *T. gondii* genotypes, particularly Type I, among liver cancer patients (Figure 3).

**Figure 4:**
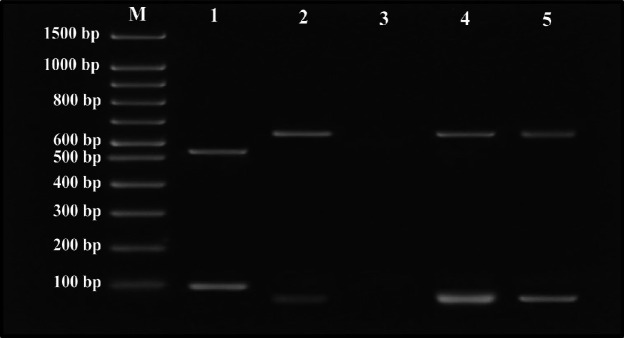
Genotypic Differentiation of *Toxoplasma gondii* in Control Group using *GRA6 *Gene RFLP Analysis. Representative gel image showing RFLP patterns of GRA6 gene amplicons from five healthy individuals after MseI digestion. Lanes (2, 4 and 5 with 75 bp, 623 bp) Type II; lane (1 with 97 bp, 544 bp) Type III; no Type I was detected. M: molecular ladder (100 bp).

The PCR amplicons of the *Toxoplasma gondii GRA6* gene from individuals in the control group were subjected to enzymatic digestion using MseI, aiming to identify the parasite’s genotypes through RFLP analysis. Type II was detected in three isolates, producing bands of approximately 75 bp and 623 bp, while Type III was observed in one isolate, yielding bands at 97 bp and 544 bp. Type I was not detected in any of the examined samples. These findings indicate the predominance of Type II among control group and reflect a limited genotypic diversity of *T. gondii* in this group, with a complete absence of Type I (Figure 4).

### Type I T. gondii strain linked to greater liver injury

Statistical analysis of liver enzyme levels (ALT, AST, ALP) in samples infected with different *Toxoplasma gondii *genotypes revealed significant differences, indicating variable hepatic impact among the strains. The Type I genotype exhibited the highest mean levels across all enzymes, with ALT at 73.47 U/L, AST at 68.12 U/L, and ALP at 188.59 U/L. These differences were statistically significant (p<0.05) when compared to results of type II and type III. The Type II strain showed intermediate enzyme levels: ALT (62.51 U/L), AST (55.33 U/L), and ALP (167.49 U/L), while the Type III genotype was associated with the lowest values: ALT (44.70 U/L), AST (39.19 U/L), and ALP (142.74 U/L). These findings highlight a clear association between *T. gondii* genotype and the degree of hepatic involvement, with Type I appearing to be the most virulent, potentially causing greater hepatocellular and biliary injury than Types II and III (Table 4).

## Discussion

The current study, as evidenced by ELISA findings, revealed a statistically significant difference in the seroprevalence of *T. gondii* between liver cancer patients (53%) and healthy individuals (35%) (p< 0.05). This indicates a potential association between hepatic disorders and increased susceptibility to *T. gondii* infection. These results align with prior research in Iraq. Such as a study by [23] reported seroprevalence rates of approximately 57.5% in liver disease patients versus 28.33% in healthy controls. Moreover, a case–control study in Baghdad found 62.85% seropositivity in chronic liver disease patients compared to 27.28% in controls [24]. Regional and international literature reinforce this trend. In Egypt, [25] documented significantly elevated rates 30% in chronic liver disease patients versus 6% in healthy controls. All of the aforementioned studies, whether local or regional, are consistent with the findings of the current study.

Our results agree with that stated by [26] who reported high seroprevalence (65.5%) of *T. gondii* antibodies in patients with acute and chronic liver diseases against a 27% seroprevalence found in the group of healthy control subjects. Our results also agreed with other investigators who stated an association of Toxoplasmosis with liver diseases [27]. On other hand our results disagreed with those illustrated by [28] who did not show any link between seropositivity to *T. gondii* and liver diseases with comparable seroprevalence of *T. gondii* IgM and IgG levels in CLD patients and control subjects.

These findings suggest a significant association between *Toxoplasma gondii *infection and impaired liver function, implying that the presence of hepatic disorders may facilitate the establishment or reactivation of the parasite due to compromised immune responses or altered physiological conditions within the liver. This increased prevalence in hepatic patients may stem from impaired immunity, altered cytokine responses, or heightened exposure to transmission risk factors such as blood transfusions, consumption of undercooked meat, or contact with contaminated water or soil. These findings emphasize the importance of routine toxoplasmosis screening among liver disease patients, especially in endemic regions.

The association of Toxoplasma and liver dysfunction confirmed in this study could have two directions: the infection with Toxoplasma increases the risk of liver injury or liver dysfunction increases the risk of *T. gondii* infection. Previous studies linked Toxoplasma infection to liver injury and its association with necrosis, hepatomegaly, granuloma and hepatitis [12]. On the other hand, CLD increases the susceptibility to Toxoplasma infection [29].

This study revealed significantly elevated liver enzyme levels in liver cancer patients co-infected with *T. gondii*, suggesting a potential synergistic or compounding effect. A longitudinal cohort in China by [30] found that *T. gondii* seropositivity was independently associated with elevated ALT/AST and was more common in individuals with metabolic liver disorders, suggesting a broader hepatic burden than previously thought. Furthermore, Our findings are supported by national survey data from the U.S. (NHANES 2009–2010), where *T. gondii*-seropositive individuals demonstrated significantly higher odds of elevated liver injury biomarkers (ALT, AST, GGT, ALP) and increased risk of both chronic liver disease and NAFLD. Further, in a preliminary analysis using the same NHANES dataset, reported that seropositive participants had a higher prevalence of combined chronic liver, kidney, and cardiovascular disease, with liver disease rates at 2.76% vs 1.26% in seronegative individual [12, 31].

Several mechanisms may explain the elevated liver enzyme levels in *T. gondii*-infected individuals. Mechanistically, *T. gondii* is known to induce a strong pro-inflammatory cytokine response, including the upregulation of IFN-γ, TNF-α, and IL-6, which may contribute to liver damage through immune-mediated pathways [32]. This inflammatory cascade could further exacerbate liver injury in infected individuals who already have underlying hepatic inflammation and fibrosis. The parasite may also exert direct cytopathic effects on hepatocytes, as demonstrated in histopathological studies [33]. Oxidative stress and cellular damage, Chronic infection with *T. gondii* results in elevated reactive oxygen species (ROS) within hepatocytes, contributing to cell lysis and leakage of liver enzymes [28].

Several genetic markers have been utilized for *T. gondii* strain typing, among these markers, *GRA6* gene is generally used for genetic characterization and typing of *T. gondii* isolated from humans, animals, and meat products [34–36]. To the best of our knowledge, there is no study on genotyping of *T. gondii* isolates from liver cancer patients using PCR-RFLP methods, in Iraq, therefore, this study was designed for the characterization and analysis of the genetic variation of *T. gondii*. In the present study, we used PCR-RFLP assay at GRA6 locus for genotyping of *T. gondii* strains isolated from liver cancer patient’s in addition healthy individuals. Many previous studies were depending upon the nested PCR-RFLP method to determine the genotypes of the *T. gondii* at GRA6 [19, 21, 37, 38].

In this study, GRA6 was used for genotyping the parasite because the coding region of this gene has considerable polymorphism, and even in comparison to other examined T. coding genes such as *SAG1*, *SAG2*, and *GRA4* is more variable, rate of amino acid changes, non-synonymous to synonymous, is high so this fact show variation in *GRA6* genes of different isolates of *T. gondii* may influence survival of the parasite particularly in the parasitophorous vacuole [19, 21]. Similarly, a recent investigation emphasized the relevance of the *GRA6* gene as a reliable genetic marker for strain differentiation, particularly in clinical samples [39]. The use of nested PCR targeting the *GRA6* gene proved to be a sensitive and reliable method for genotyping. The *GRA6* gene’s high polymorphism has made it a widely used marker for strain differentiation, especially when combined with RFLP analysis [40].

Several biological mechanisms may explain the detection of GRA6 in all tested samples: 1. Latent infection: *T. gondii* forms tissue cysts that remain dormant in immunocompetent hosts, particularly in organs like the liver and brain, 2. Immunosuppression: Chronic liver diseases such as HBV and HCV impair immune function, allowing parasite proliferation or reactivation, 3. Environmental burden: Positive results among healthy individuals may reflect community-wide exposure through contaminated food, water, or zoonotic sources and 4. High diagnostic sensitivity: Nested PCR targeting the *GRA6* gene allows for detection of low parasitic DNA loads that would be missed by conventional techniques.

Our use of the *GRA6* gene and MseI digestion for genotyping provides robust discrimination among the clonal lineages. The polymorphic nature of GRA6 and the specificity of MseI restriction sites allow precise genotype differentiation, lending credibility to the reported genotype distribution.

The present study reported Type I as the dominant *Toxoplasma gondii *genotype in liver cancer patients and it consistent with some studies conducted in other geographic regions have reported contrasting patterns, with a predominance of Type I, Type III, or atypical genotypes. For example, [41] reported a high prevalence of Type I strains among clinical and congenital toxoplasmosis cases in Brazil. Their study emphasized the increased virulence and severity of disease outcomes associated with Type I strains in South America, especially among immunocompromised individuals and fetuses. Similarly, through a multilocus PCR-RFLP genotyping approach, the study documented the circulation of a diverse set of non-clonal and hybrid strains across Central and South America [42]. Their findings highlight that the global *T. gondii* population structure is not uniform and that regional ecological and host factors may drive the emergence of distinct genetic lineages.

In Eastern Europe, identified Type III as the most frequent genotype among human toxoplasmosis cases in Serbia, contrasting with the dominance of Type II reported in Western Europe [43]. This variation was suggested to result from different exposure sources and environmental reservoirs. Moreover, a study analyzed cerebrospinal fluid samples from AIDS patients with toxoplasmic encephalitis in Cuba and revealed a predominance of Type I and recombinant strains [44]. The authors speculated that Type I strains may have greater neurotropism and are more likely to cause reactivation in immunocompromised hosts.

On the other hand, the predominant genotype identified across healthy group was Type II. These findings contribute valuable insights into the epidemiology of *T. gondii* in patients with hepatic pathologies and healthy controls within the study region. Further regional data are consistent with our study such as study by [45] reported significant predominance of Type II in Iranian patients with immune and hepatic disorders, reinforcing the genotype’s ability to establish persistent infection in vulnerable hosts. Another study showed that Genotype II was found in 29% of isolates from congenital toxoplasmosis patients in Brazil, indicating the genotype’s high frequency in the afflicted community [46]. Likewise, 78.57% of human *Toxoplasma gondii *isolates in China were of Genotype II, highlighting its regional dominance [8]. Numerous genotyping investigations conducted in Europe have further supported this pattern, showing that the prevalence of Genotype II in clinical human samples exceeds 85% [47].

These results are consistent with our recent study and indicate a regional pattern in the Middle East favoring Type II dominance, which may be influenced by host genetics, environmental factors, or parasite adaptation mechanisms. The predominance of Type II *T. gondii* in healthy group can be attributed to its intermediate virulence. Unlike the highly virulent Type I strains that seen in liver cancer patients, who often cause acute, sometimes lethal infections and might be underrepresented in chronic disease cohorts, Type II strikes a balance that favors persistence and transmission. Type III strains, while less virulent, are less commonly isolated in human infections globally and appear less adapted to long-term persistence in humans.

Additionally, the frequent detection of Type I in liver cancer patients raises questions about its potential role as a cofactor in carcinogenesis through chronic inflammation or immune modulation [48].

These discrepancies can be attributed to several factors. Firstly, the geographic location plays a pivotal role in the distribution of *T. gondii* genotypes. In Latin America, especially Brazil and Mexico, the presence of wild feline and avian reservoirs supports the circulation of genetically diverse and virulent strains, unlike the more clonal populations observed in Europe and the Middle East [42]. Secondly, host immunity and clinical manifestation influence strain detection; studies focusing on acute or neuroinvasive toxoplasmosis are more likely to isolate highly virulent strains such as Type I. Thirdly, the diagnostic technique matters: while this study used GRA6-based nested PCR and MseI digestion for genotyping, others employed multilocus or whole-genome sequencing, which enables more precise identification of hybrid and atypical lineages [49]. These findings highlight the importance of molecular genotyping not only for epidemiological surveillance but also for understanding strain-specific pathogenicity and its interaction with host immune status.

In conclusion, the predominance of genotype I among Liver cancer patients highlights the genetic diversity of *T. gondii* and it may contribute to the progression or severity of liver cancer, as evidenced by a significant increase in liver enzyme levels among co-infected patients. These findings are significant for understanding the genetic distribution of *T. gondii* in the region and underscore the need for future research and molecular surveillance to explore the relationship between parasite genotypes and chronic liver diseases.

**Table 1:** Prevalence of *Toxoplasma Gondii *in Liver Cancer Patients & Control

Study groups	Total cases	Positive Toxoplasma Igs		Negative Toxoplasma Igs	
		No.	%	No.	%
Liver cancer	40	21	53	19	47
Control	40	14	35	26	65

p value = 0.001**

**Table 2:** Liver Enzyme Levels in Liver Cancer Patients with and without Toxoplasma Infection

Parameters	Liver cancer patients			Healthy group		
	Toxo +ve (mean±SD)	Toxo -ve (mean±SD)	p-value	Toxo +ve (mean±SD)	Toxo -ve (mean±SD)	p-value
ALT	76.370±15.671	67.010±12.837	0.029*	28.584±6.552	18.313±7.833	0.008*
AST	82.710±11.042	70.410±8.346	0.000*	23.100±4.563	15.930±6.202	0.008*
ALP	183.756±6.760	167.139±15.134	0.003*	78.261±10.866	64.204±15.143	0.007*

**Table 3:** Nested-PCR Results and RFLP Patterns for *GRA6* Gene

Sample No.	Disease Type	Nested-PCR Result	Genotype (MseI digestion)	Fragment Sizes (bp)
1	Liver Cancer	Positive	Type I	168 bp, 544 bp
2	Liver Cancer	Positive	Type I	168 bp, 544 bp
3	Liver Cancer	Positive	Type III	97 bp, 544 bp
4	Healthy Control	Positive	Type II	75 bp, 623 bp
5	Healthy Control	Positive	Type II	75 bp, 623 bp
6	Healthy Control	Positive	Type II	75 bp, 623 bp
7	Healthy Control	Positive	Type III	97 bp, 544 bp

**Table 4:** Effect of Genotype of* T. gondii* on Liver Enzyme Levels

Parameters	Genotypes			
	Type I Mean±SD	Type II Mean±SD	Type III Mean±SD	p value
ALT	73.47±23.94	62.51±17.22	44.70±11.61	0.002**
AST	68.12±20.27	55.33±15.91	39.19±9.07	0.001**
ALP	188.59±13.52	167.49±14.10	142.74±11.69	0.001**

## Author Contribution Statement

All authors had equal roles in design, work, statistical analysis, and manuscript writing.
